# Near-Field Beam Training in Terahertz Communications with Hybrid Beamforming Architecture

**DOI:** 10.3390/mi14040880

**Published:** 2023-04-19

**Authors:** Yuxin Xie, Boyu Ning, Lingxiang Li, Zhi Chen

**Affiliations:** The National Key Laboratory on Wireless Communications, University of Electronic Science and Technology of China, Chengdu 611731, China

**Keywords:** terahertz communication, near-field, hybrid beamforming, beam training, tangent arrangement

## Abstract

Terahertz (THz) communication has a large available bandwidth, which is expected to be deployed in future communication networks. As THz wave suffers from severe propagation loss in wireless transmission, we consider a THz near-field scenario where a base station (BS) is equipped with a large-scale antenna array with a low-cost hybrid beamforming architecture to serve mobile users nearby. However, the large-scale array and the user mobility incur difficulty in channel estimation. To tackle this issue, we propose a near-field beam training scheme that can align a beam to the user in a fast way by searching the codebook. Specifically, the BS employs a uniform circular array (UCA), and the radiation pattern of the beams in our proposed codebook appears as ellipsoids. To cover the serving zone with the minimum codebook size, we develop a near-field codebook by tangent arrangement approach (TAA). To reduce the time overhead, we leverage the hybrid beamforming architecture to realize multi-beam training concurrently since each RF chain can enable a codeword whose element has a constant magnitude. Numerical results validate that our proposed UCA near-field codebook achieves less time cost while achieving a comparable coverage performance compared to the conventional near-field codebook.

## 1. Introduction

With the increasing demand for higher bandwidth and higher data rates, Terahertz (THz) is envisioned as a key band for ultra-wideband wireless systems that support six-generation (6G) mobile systems [1]. This is owing to that THz communication technology can significantly mitigate the problem of spectrum scarcity, greatly improves the channel capacity, and meet the needs of emerging new applications such as augmented reality (AR), visual reality (VR), and connected autonomous systems [2]. The authors in [3,4] provide a comprehensive view of end-to-end 6G THz communication systems, highlighting key advancements and opportunities for the physical, link, and network layers.

However, due to the high frequency in the THz band, the THz signal inherently suffers from severe propagation loss, line-of-sight (LoS) blockage, and the effect of molecular absorption. In this context, beamforming technology under the THz ultra-massive multiple-input-multiple-output (UM-MIMO) is envisioned as a key technology for future THz wireless systems [5,6]. Meanwhile, owing to the location of a moving user that can hardly be accurately estimated, beam training is known as a significant beamforming solution with predefined beams in a codebook that does not need for the channel estimation process [7].

Specifically, based on the codebook, beam training scans the predefined beams to search the user without the channel state information (CSI) [7,8]. Commonly, single-beam training that switches the codewords one by one is employed to determine the optimal beam pair. This scheme has been widely used in radar systems with phased arrays since there is only one digital input/output (IO) for all antennas, which can merely identify one beam at a time. The hardware cost of single-beam scanning is relatively low, but it may not meet the real-time communication requirements under a large codebook size due to its time-consuming manner [9].

To accelerate the training process, it is viable to use a fully digital architecture in which each antenna corresponds to an independent radio frequency (RF) chain to transmit or detect multiple beams concurrently. However, UM-MIMO systems using digital beamforming require a large number of RF chains, leading to high hardware costs and computational overhead. Therefore, a hybrid (digital and analog) beamforming architecture can be adopted to reduce total overhead [10,11]. To be exact, the hybrid beamforming architecture uses fewer RF chains than antennas, each RF chain connected to all antennas via phase shifters (called analog beamforming) to balance the performance and cost [12].

For a near-field communication scenario, the region boundary, or maximum radius, is usually determined by the Rayleigh formula, i.e., $2D^{2}/\lambda$, where *D* is the array size and λ is the wavelength [13]. For typical wireless systems, such as an array size of 0.1 m with an operation frequency of 30 GHz, the Rayleigh distance is about 2 m. However, this distance grows to approximately 22.7 m at 0.34 THz. Therefore, in the THz communication scenario, more users might fall in the near-field communication region, and the conventional far-field beam training scheme is no longer applicable [14].

There have been some works studying near-field beam training. For example, the authors in [15] established an analytically range-angle dependent beam focusing model for THz linear antenna arrays, uncovering that the achievable focusing spatial region constitutes a rotated ellipse centered at the target. The authors in [16,17] considered the design of the near-field codebook by replacing the classical representation in the angular domain with one in the polar domain. The authors in [18] proposed a spherical-domain sampling method for designing the uniform planar array (UPA) codebook. The authors in [19] considers a wideband OFDM THz MIMO architecture of ULA. However, it should be noted that the near-field beam training schemes mentioned above are all suited for uniform linear arrays (ULAs) or UPAs. The authors in [20] firstly proposed that compared with ULA, the uniform circular array (UCA) is more suitable for near-field beam training. This is because the UCA’s beam near-field beam pattern tends to be a far-field one when the elevation angle tends to be 0, which makes it possible to reduce the codebook size and accelerate the training process. Moreover, the rotational symmetry of UCA provides a uniform array gain at any azimuth angles [21,22]. Although the codebook given in [20] can cover the whole target serving zone, it has some drawbacks, such as the inaccurate characterization of the beam’s coverage boundary and high time complexity based on the single-beam training.

To fix these issues, in this paper, we propose a multi-beam near-field beam training scheme in THz UCA by using hybrid beamforming architecture. The main contributions are summarized as follows.

1. We establish a near-field communication model for THz UCA with hybrid beamformer architecture. Based on the power of one beam in another beam’s focusing direction, we define the beam coherence function between two beams with different directions in the three-dimensional (3D) space.
2. By analyzing the length, width, and thickness of the beam shape, which can be proved to be an ellipsoid, we propose a new codebook design for the near-field UCA by determining the 3D codewords’ parameters of three different domains successively. Specifically, we propose a tangent arrangement approach (TAA) to design the codewords’ parameters to arrange the beams in the near-field region reasonably. This way, each beam is tangent to one to balance the performance and codebook size.
3. To further reduce the training time cost, we propose a multi-beam training scheme thanks to the hybrid beamforming architecture adopted. Simulation results show that our proposed TAA and multi-beam training scheme can accelerate the beam training while achieving comparable coverage performance compared to the ULA codebook given in [16] and the UCA codebook given in [20].

## 2. System Model and Channel Model

**Figure 1:**
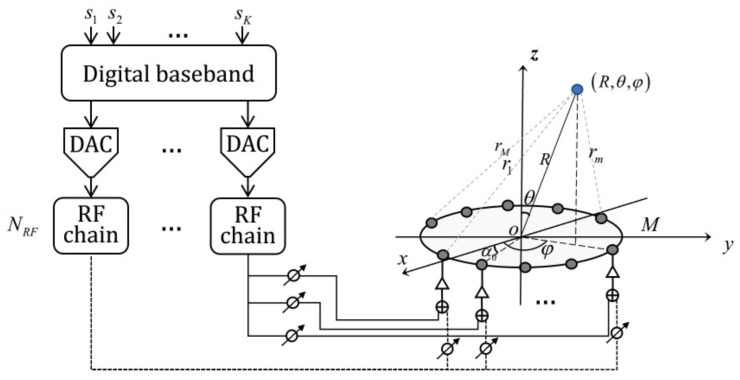
Illustration of THz UCA near-field beamforming approach.

We consider the downlink transmission the downlink transmission from a THz transmitter equipped with a hybrid beamforming architecture comprising of an *M*-antenna UCA to a single-antenna user in the near-field region, as illustrated in Figure 1.

### 2.1. System Model

A hybrid beamformer consists of a baseband precoder $F_{B}\in C^{N_{RF}\times K}$ connected through $N_{RF}$ RF chains to the RF beamformer $F_{RF}\in C^{M\times N_{RF}}$ such that $F=F_{RF}F_{B}$. Since the phase shifters in the analog precoder can only change the phase of the transmitted signal, each entry of $F_{RF}$ is constrained by the same amplitude, which can be written as

(1) $$F_{RF}=\left(\begin{array}{cccc} e^{j\rho_{11}} & e^{j\rho_{12}} & \cdots & e^{j\rho_{1N_{RF}}}\\ e^{j\rho_{21}} & e^{j\rho_{22}} & \cdots & e^{j\rho_{2N_{RF}}}\\ \vdots & \vdots & \ddots & \vdots \\ e^{j\rho_{M1}} & e^{j\rho_{M2}} & \cdots & e^{j\rho_{1MN_{RF}}} \end{array}\right)$$

where $\forall \rho_{mn}\in \Theta ,m\in \left\{1,\cdots ,M\right\},n\in \left\{1,\cdots ,N_{RF}\right\}$ and Θ∈[0,2π] for analog phase shifters.

The transmitter sends a symbol vector $s\in C^{K\times 1}$ to the target user, which is assumed to satisfy $E\left\{ss^{H}\right\}=I_{K}$. To meet the total transmitted power constraint, $F_{B}$ is normalized to satisfy ${∥F_{RF}F_{B}∥}_{F}^{2}=1$. Then the transmitted signal can be written as:

(2) x=FPs

where $P\in R^{K\times K}$ is a diagonal power allocation matrix satisfying ${∥P∥}_{F}^{2}=P_{t}$ and $P_{t}$ is the total transmit power.

The received signal *y* can be expressed as:

(3) $$y=h^{H}x+n=h^{H}FPs+n$$

where $h≜{\left[h_{1},\cdots ,h_{M}\right]}^{T}$ denotes the wireless channel, and *n* denote noise.

### 2.2. Channel Model

The target user locates at point D(R,θ,φ), i.e., (Rsinθcosφ,Rsinθcosφ,Rcosθ) in Cartesian coordinate system, where R,θ,φ represent the distance (to the center of UCA), elevation angle and azimuth angle, respectively. The location of the *m*-th antenna of UCA with a radius of $r_{0}$ is denoted by $\left(r_{0},\pi /2,\alpha_{m}\right)$, where $\alpha_{m}=\left(m-1\right)\alpha_{0}$ and $\alpha_{0}=2\pi /M$ is the angle between two adjacent antennas.

Consider THz spherical wave model, the propagation distance from the *m*-th antenna of UCA to the user is

(4) $$\begin{array}{cc} r_{m}\left(R,\theta ,\varphi \right) & ={\left\{{\left[R\sin\theta \cos\varphi -r_{0}\cos\alpha_{m}\right]}^{2}+{\left[R\sin\theta \sin\varphi -r_{0}\sin\alpha_{m}\right]}^{2}+{\left(R\cos\theta \right)}^{2}\right\}}^{1/2}\\ \; & ={\left\{R^{2}+r_{0}^{2}-2Rr_{0}\sin\theta \cos\left(\varphi -\alpha_{m}\right)\right\}}^{1/2}\\ \; & \overset{\left(a\right)}{\approx }R-r_{0}\sin\theta \cos\left(\varphi -\alpha_{m}\right)+\frac{r_{0}^{2}\left[1-\sin^{2}\theta \cos^{2}\left(\varphi -\alpha_{m}\right)\right]}{2R} \end{array}$$

where the approximation (a) is derived from the second-order Taylor series expansion, $\sqrt{1+x}=1+x/2-x^{2}/8+O\left(x^{3}\right)$, assuming *R* is large compared to the other items.

In THz narrow-band communication, owing to the strong absorption level of atmospheric gases, the signal suffers from spreading loss and molecular absorption loss. According to [23], we have

(5) $$l_{Spr}\left(f,d\right)=\frac{c}{4\pi fd}$$

(6) $$l_{Abs}\left(f,d\right)=e^{-\frac{\kappa (f)fd}{2}}$$

where *f* denote the carrier frequency, *d* denote the spread distance, $l_{Spr}\left(f,r_{m}\right)$ is the spreading loss, $l_{Abs}\left(f,r_{m}\right)$ is the molecular absorption loss, and κ(f) is the frequency-dependent medium absorption coefficient.

Usually, the operation frequency band is chosen in the THz transmission window, where the molecular absorption loss of each frequency point is basically the same and relatively small, considering the relatively dry environment. In addition, we assume that the difference in path loss from antenna elements to the target user can be ignored since the difference in $r_{m}$s is comparable to the wavelength of THz; it is tiny in absolute value.

So the total loss from UCA to the user denotes $l\left(f,R\right)=l_{Spr}\left(f,R\right)l_{Abs}\left(f,R\right)$, which is the same for different antennas. Then the wireless channel can be written as:

(7) $$h\left(f;R,\theta ,\varphi \right)=\sqrt{M}l\left(f,R\right)a\left(f;R,\theta ,\varphi \right)$$

where a(f;R,θ,φ) denotes the normalized array response vector, i.e., the phase change of each antenna to the user. Specifically, for UCA with *M*-elements, the normalized spherical-wave array response vector is given by

(8) $$a\left(f;R,\theta ,\varphi \right)=\frac{1}{\sqrt{M}}{\left[e^{-j\frac{2\pi f}{c}r_{1}},\cdots ,e^{-j\frac{2\pi f}{c}r_{M}}\right]}^{T}$$

Then the received signal *y* of the frequency *f* at point D(R,θ,φ) can be written as:

(9) $$y=h^{H}x+n=\sqrt{M}la^{H}F_{RF}F_{B}Ps+n$$

## 3. Problem Description

In the actual communication scenario, it is impracticable to solve the real-time beamforming solution since the location of a moving user can hardly be accurately estimated. As the line-of-sight (LoS) propagation component is dominant in THz channels [24], beam training is known as an attractive low-complexity beamforming strategy, which predefines beams in a codebook whose radiation concentration covers all possible locations of the users [7]. Then beam training scans the beam to establish communication without CSI.

To reduce the complexity of digital beamforming, requiring control at each antenna, the hybrid approach uses two-stage beamforming (the concatenation of analog and digital beamforming) and provides a reasonable compromise between performance and complexity. Hybrid beamformers provide a limited multi-beam capability, which can significantly accelerate beam training in the near-field scenario.

Consider hybrid beamforming, let $K=N_{RF}$, $N_{RF}$ RF chains can generate $N_{RF}$ beams. We define $\omega_{n}={\left[e^{j\rho_{1n}},\cdots ,e^{j\rho_{Mn}}\right]}^{T}$, where $\rho_{mn}$ represents the phase of the phase shifter of the *m*-th antenna and $n\in \{1,\cdots ,N_{RF}\}$. Then the matrix in Formula (1) can be written as $F_{RF}=\left[\omega_{1},\cdots ,\omega_{N_{RF}}\right]$, where a column of the matrix $\omega_{n}$ is the analog beamforming vector of the *n*th RF chain.

Then, with $\omega_{n}$, the normalized signal power *B* received at the user of the *n*th RF chain is the square of the inner product of $\omega_{n}$ and a:

(10) $$\begin{array}{cc} B(f;R,\theta ,\varphi |\omega_{n}) & ={\left|\omega_{n}^{H}\cdot a\left(f;R,\theta ,\varphi \right)\right|}^{2}\\ \; & =\frac{1}{M}{\left|\sum_{m=1}^{M}e^{j\left(\frac{2\pi f}{c}r_{m}\left(R,\theta ,\varphi \right)-\beta_{mn}\right)}\right|}^{2} \end{array}$$

To maximize the received power at point D(R,θ,φ), we define $\beta_{mn}=\frac{2\pi f}{c}r_{m}^{D}$ where $r_{m}^{D}≜r_{m}\left(R,\theta ,\varphi \right)$. So that the signal power *B* can be written as:

(11) $$B\left(f;R,\theta ,\varphi |\omega_{n}\right)=\frac{1}{M}{\left|\sum_{m=1}^{M}e^{j\frac{2\pi f}{c}\left(r_{m}\left(R,\theta ,\varphi \right)-r_{m}^{D}\left(R,\theta ,\varphi \right)\right)}\right|}^{2}$$

According to the concept of the power of one beam in another beam’s focusing direction, we define the beam coherence between two beams with different directions in 3D space. In other words, set $D_{1}\left(R_{1},\theta_{1},\varphi_{1}\right)$ and $D_{2}\left(R_{2},\theta_{2},\varphi_{2}\right)$, the beam coherence is the received signal power at point $D_{2}$ with the target user at point $D_{1}$. For the convenience of analysis, we ignore the square, so the beam coherence can be written as

(12) ff;R1,R2;θ1,θ2;φ1,φ2=a(R1,θ1,φ1)Ha(R2,θ2,φ2)=1M∑m=1Mej2πfcrmD1−rmD2=(a)1M∑m=1Mej2πfcR1−R2−r0sinθ1cosφ1−αm−r0sinθ2cosφ2−αm+r021−sin2θ1cos2φ1−αm2R1−r021−sin2θ2cos2φ2−αm2R2≈1M∑m=1Mej2πfcr0sinθ1cosφ1−αm−r0sinθ2cosφ2−αm+r021−sin2θ1cos2φ1−αm2R1−r021−sin2θ2cos2φ2−αm2R2

where the approximation (a) is due to the constant value of the distance subtraction term $R_{1}-R_{2}$ and does not affect the beam coherence.

To design a multi-beam training scheme, we first predefine an excellent codebook whose radiation concentrations can cover the user’s area, i.e., all possible locations of the user, by the beam coherence in Formula (12). Then by leveraging the hybrid beamforming architecture, we develop a concrete scheme of multi-beam training based on the codebook to reduce the training time cost.

## 4. Codebook Design

In this section, we first give the steps of codebook design, i.e., the design sequence of R,θ,φ domain based on the geometric beam-shape model of UCA in [20]. Then the codewords of the target serving zone can be obtained by the beam coherence in order.

### 4.1. Codebook Design Steps

The form of Formula (12) is too complex that it is impracticable to find the codebook design scheme of R,θ,φ. Ref. [20] proposed an approximately explicit model for the near-field beam shape of UCA, which uncovers that boundary of the near-field pattern in R−θ−φ domain is an ellipsoid. The ellipsoid occupies a certain range in the three dimensions of space.

**Figure 2:**
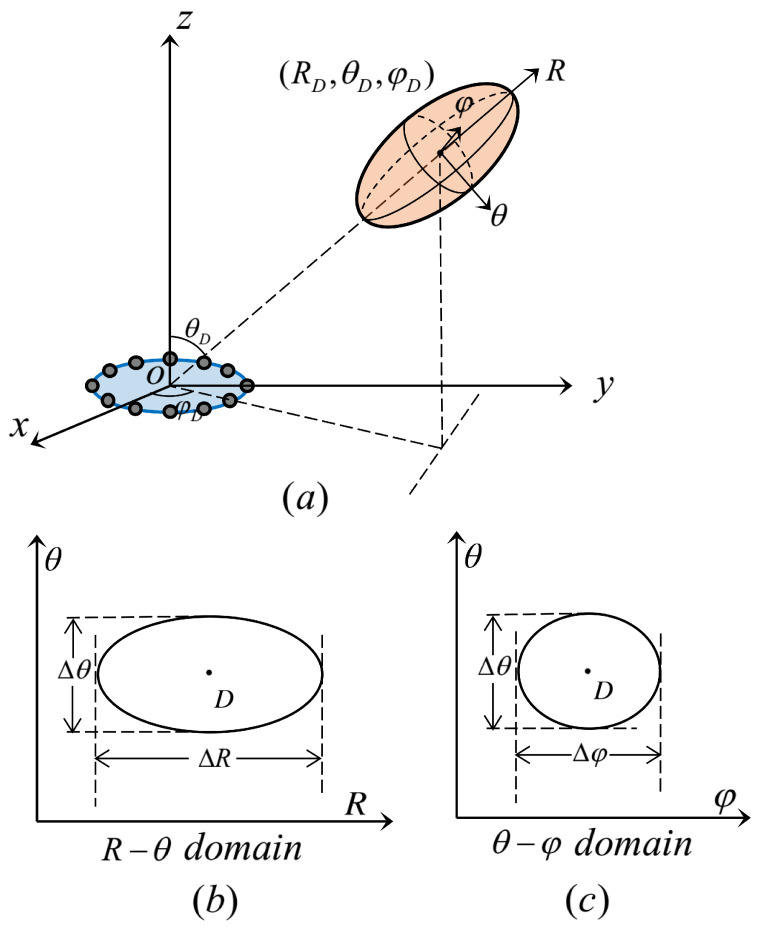
Illustration of THz UCA near-field beam pattern. (**a**) Schematic diagram of near-field focusing effect in 3D space. (**b**) Schematic diagram of beam pattern in R−θ domain. (**c**) Schematic diagram of beam pattern in θ−φ domain.

To better illustrate the geometric shape, we define the maximum difference in the coverage of the three dimensions as the length ΔR, width Δθ, and thickness Δφ of the ellipsoid. For ease of understanding, Figure 2 shows the illustration of THz UCA near-field beam pattern.

The details of the relationship between the beam pattern and point D(R,θ,φ) are summarized in Table 1 (↑ indicates an increase in the value, while ↓ indicates a decrease).

Based on Table 1, we can obtain some interesting properties as follows: (1) Δθ is only related to $\theta_{D}$; (2) Δφ is only related to $\theta_{D}$; (3) ΔR is only related to $\theta_{D}$ and $R_{D}$.

So we propose a codebook design steps of near-field UCA: first design θ domain, then design φ domain, and finally design *R* domain. The reasons are as follows:

- The purpose of designing θ domain is to obtain the $\theta_{D}$-selectable set that can cover the whole communication serving zone. In addition to the initial beam, $\theta_{D}$ of each beam is determined in conjunction with $\theta_{D}$ and Δθ of the previous beam. The reason for designing θ domain in the first step is that Δθ is only related to $\theta_{D}$ in its domain, not to $R_{D},\varphi_{D}$. Therefore, only θ domain can be designed independently, while the other two domains are not designed.
- Then, we design φ domain, i.e., obtain $\varphi_{D}$ of different beams by Δφ. The reason for designing φ domain in step 2 is because that Δφ is only related to $\theta_{D}$, and $\theta_{D}$-selectable set has been determined in the first step.
- Finally, we designed *R* domain, i.e., obtain $R_{D}$ of different beams by ΔR. It must be designed after θ domain since ΔR is determined by $\theta_{D}$ and $R_{D}$ together. In addition, for different $\theta_{D}$, there are different *R*-selectable sets. It is worth mentioning that the design of *R* domain is not affected by φ domain and vice-versa. So step 2 and step 3 are interchangeable.

Next, we give the specific design scheme of θ,φ,R domain in order.

#### 4.1.1. θ Domain

**Figure 3:**
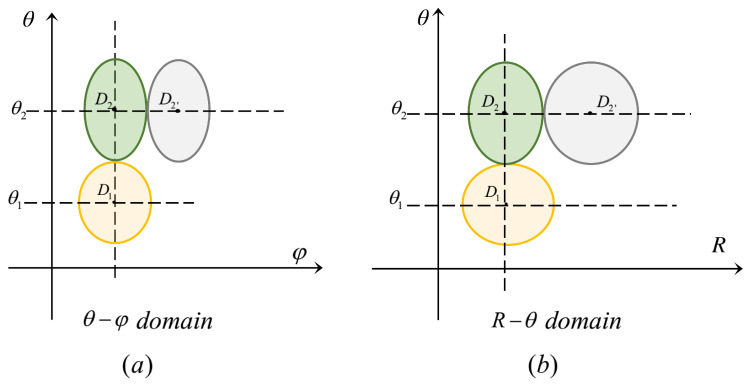
Illustration of beam patterns. (**a**) Beam arrangement in θ−φ domain. (**b**) Beam arrangement in R−θ domain.

We define the *n*-th designed angle: $\theta_{n}\overset{\Delta }{\;=}\theta_{D_{n}}$. Given that the angle of the initial beam is $\theta_{1}$, we should consider the nearest beam in designing $\theta_{2}$ and calculate the coherence between the two beams. It is obvious that the distance is closest when $R_{D}$ and $\varphi_{D}$ are equal. Figure 3 plots beam patterns layout (The beam in yellow represents the reference beam of $\theta_{1}$, the beam in green represents the nearest beam, and the beam in grey represents the non-nearest beam):

As shown in the left figure above, we can see that the initial angle of beam $D_{1}$ is $\theta_{1}$. When choosing $\theta_{2}$, for beams $D_{2}$ and $D_{2^{\prime }}$ of $\theta_{2}$, we should consider the nearest beam $D_{2}$, i.e., the beam whose $\varphi_{D}$ is equal to $D_{1}$. In the same way, for beam $D_{1}$ as shown in Figure 3b, choose the nearest beam $D_{2}$, i.e., the beam $D_{2}$ whose $R_{D}$ is equal to $D_{1}$, rather than $D_{2^{\prime }}$.

Therefore, we let $R_{1}=R_{2}=R_{0}$ and $\varphi_{1}=\varphi_{2}=\varphi_{0}$, the beam coherence can be expressed as follows:

(13) $$f\left(f;R_{0},R_{0};\theta_{1},\theta_{2};\varphi_{0},\varphi_{0}\right)=\left|\frac{1}{M}\sum_{m=1}^{M}e^{j\frac{2\pi f}{c}\left[r_{0}\sin\theta_{2}\cos\left(\varphi_{0}-\alpha_{m}\right)-r_{0}\sin\theta_{1}\cos\left(\varphi_{0}-\alpha_{m}\right)\right]}\right|$$

Let $x_{\theta }=\frac{2\pi f}{c}r_{0}\left(\sin\theta_{2}-\sin\theta_{1}\right)$, we define the θ-coherence:

(14) $$f_{\theta }\left(x_{\theta }\right)=\left|\frac{1}{M}\sum_{m=1}^{M}e^{j\cos t_{m}x_{\theta }}\right|$$

where $t_{m}=\varphi_{0}-\alpha_{m}$ and $t_{m}\in \left[0,2\pi \right]$.

**Discussion** **1.** *In THz communications, the number of antennas in massive MIMO can be much larger than that for MIMO in 5G communication. So $\alpha_{0}=\frac{2\pi }{M}$ is minimal that $t_{m}$ can be approximately continuous in [0,2π]. So Equation (14) can be presented as:*

(15) $$f_{\theta }\left(x_{\theta }\right)\approx \left|\frac{1}{2\pi }\int_{0}^{2\pi }e^{j\cos t_{m}x_{\theta }}dt_{m}\right|$$

*The Bessel function of the first kind is known as*

$$J_{n}\left(x\right)=\frac{1}{\pi }\int_{0}^{\pi }\cos(nt-x\sin t)dt=\frac{1}{2\pi }\int_{0}^{2\pi }e^{j(nt-x\sin t)}dt$$

*We can find that Equation (15) conforms to the zero-order Bessel function of the first kind.*

#### 4.1.2. φ Domain

We already have the $\theta_{D}$-selectable set in the θ domain. Since Δφ is only related to $\theta_{D}$, beams with equal $R_{D}$ and $\theta_{D}$ are equally distributed in the form of arithmetic progression (average in φ domain). Set averaging degree coefficient: $x_{\varphi }^{n}\left(\theta_{n}\right)$. As shown in Figure 3a, two adjacent beams $D_{2}$ and $D_{2^{\prime }}$ in $\theta_{2}$ should be considered when calculate the φ domain averaging degree coefficient $x_{\varphi }^{2}\left(\theta_{2}\right)$.

Therefore, we let $R_{1}=R_{2}=R_{0}$ and $\theta_{1}=\theta_{2}=\theta_{0}$, the beam coherence can be expressed as follows:

(16) $$\begin{array}{cc} \; & f_{\varphi }\left(f;R_{0},R_{0};\theta_{n},\theta_{n};\varphi_{1},\varphi_{2}\right)\\ \; & =\left|\frac{1}{M}\sum_{m=1}^{M}e^{j\frac{2\pi f}{c}\left\{r_{0}\sin\theta_{n}\left[\cos\left(\varphi_{{}_{2}}-\alpha_{m}\right)-\cos\left(\varphi_{1}-\alpha_{m}\right)\right]+\frac{r_{0}^{2}}{2R_{0}}\sin^{2}\theta_{n}\left[\cos^{2}\left(\varphi_{2}-\alpha_{m}\right)-\cos^{2}\left(\varphi_{1}-\alpha_{m}\right)\right]\right\}}\right| \end{array}$$

Since the value of $R_{0}$ does not affect the result of the above formula, let $R_{0}=\infty$, the φ-coherence:

(17) $$f_{\varphi }\left(x_{\varphi }^{n}|\theta_{n}\right)=\left|\frac{1}{M}\sum_{m=1}^{M}e^{j\frac{2\pi f}{c}\left\{r_{0}\sin\theta_{0}\left[\cos\left(x_{\varphi }^{n}-\alpha_{m}\right)-\cos\left(\alpha_{m}\right)\right]\right\}}\right|$$

where $\varphi_{2}=\varphi_{1}+x_{\varphi }^{n}\left(\theta_{n}\right)$.

#### 4.1.3. *R* Domain

We already have the $\theta_{D}$-selectable set in θ domain. Since ΔR is independent of $\varphi_{D}$, the design of beams with equal $\varphi_{D}$ and $\theta_{D}$ is determined by $R_{D}$. Similar to the design of θ and φ, two adjacent beams with the nearest distance should be considered. As shown in Figure 3b, two adjacent beams $D_{2}$ and $D_{2^{\prime }}$ in $\theta_{2}$ should be considered.

Therefore, let $\theta_{1}=\theta_{2}=\theta_{0}$ and $\varphi_{1}=\varphi_{2}=\varphi_{0}$, the beam coherence can be expressed as follows:

(18) $$f_{R}\left(f;R_{1},R_{2};\theta_{n},\theta_{n};\varphi_{0},\varphi_{0}\right)=\left|\frac{1}{M}\sum_{m=1}^{M}e^{j\frac{2\pi }{\lambda }\left\{\frac{r_{0}^{2}\left[1-\sin^{2}\theta_{n}\cos^{2}\left(\varphi_{0}-\alpha_{m}\right)\right]}{2R_{1}}-\frac{r_{0}^{2}\left[1-\sin^{2}\theta_{n}\cos^{2}\left(\varphi_{0}-\alpha_{m}\right)\right]}{2R_{2}}\right\}}\right|$$

Let $t_{m}=\varphi_{0}-\alpha_{m}$ and $t_{m}\in \left[0,2\pi \right]$,

(19) $$\begin{array}{cc} f_{R}\left(f;R_{1},R_{2}|\theta_{n}\right) & =\left|\frac{1}{M}\sum_{m=1}^{M}e^{j\frac{\pi f}{c}r_{0}^{2}\left(\frac{1}{R_{1}}-\frac{1}{R_{2}}\right)\left[1-\sin^{2}\theta_{n}\cos^{2}t_{m}\right]}\right|\\ \; & \overset{\left(a\right)}{\approx }\left|\frac{1}{M}\sum_{m=1}^{M}e^{-j\frac{\pi f}{c}r_{0}^{2}\left(\frac{1}{R_{1}}-\frac{1}{R_{2}}\right)\sin^{2}\theta_{n}\cos^{2}t_{m}}\right| \end{array}$$

where the approximation (a) is due to the constant value of $\frac{\pi fr_{0}^{2}}{c}\left(\frac{1}{R_{1}}-\frac{1}{R_{2}}\right)$ and has no effect on the coherence.

We define the distance coefficient $x_{R}^{n}\left(f;\theta_{n}\right)=\frac{\pi fr_{0}^{2}}{c}\left(\frac{1}{R_{1}}-\frac{1}{R_{2}}\right)\sin^{2}\theta_{n}$, then the *R*-coherence:

(20) $$f_{R}\left(x_{R}^{n}|\theta_{n}\right)=\left|\frac{1}{M}\sum_{m=1}^{M}e^{-jx_{R}^{n}\cos^{2}t_{m}}\right|$$

### 4.2. Tangent Arrangement Approach

In this section, since the geometric shape of the near-field beam pattern of THz UCA is an ellipsoid, we propose TAA design a codebook to balance the coverage performance and codebook size.

**Figure 4:**
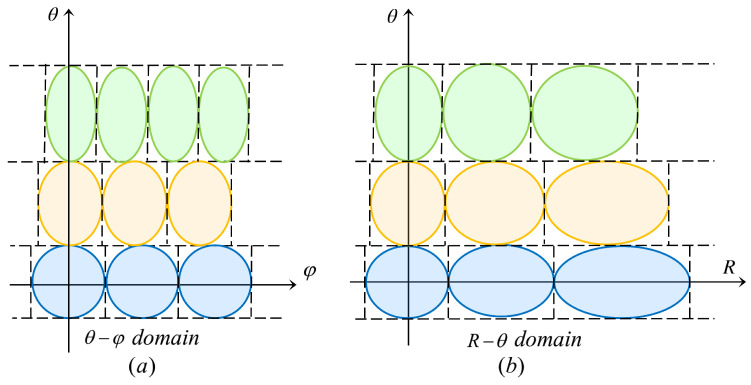
Illustration of the TAA. (**a**) θ−φ domain. (**b**) R−θ domain.

For the target serving zone $R\in \left[R_{min},R_{max}\right],\theta \in \left[\theta_{min},\theta_{max}\right],\varphi \in \left[0,2\pi \right]$, design the codebook according to the codebook design steps: θ→φ→R and the beam coherence functions in the previous section. Figure 4 plots the illustration of the TAA in θ−φ domain and R−θ domain.

To cover the whole communication serving zone with a relatively small codebook size, each beam ellipsoid must be tangent to the surrounding ellipsoid. We already know that the design of *R* domain is independent of φ domain and vice versa. So if we can guarantee that the ellipses are tangent in the 2D domain (θ−φ domain and R−θ domain), then the ellipsoid must be tangent in the 3D domain (R−θ−φ domain).

**Figure 5:**
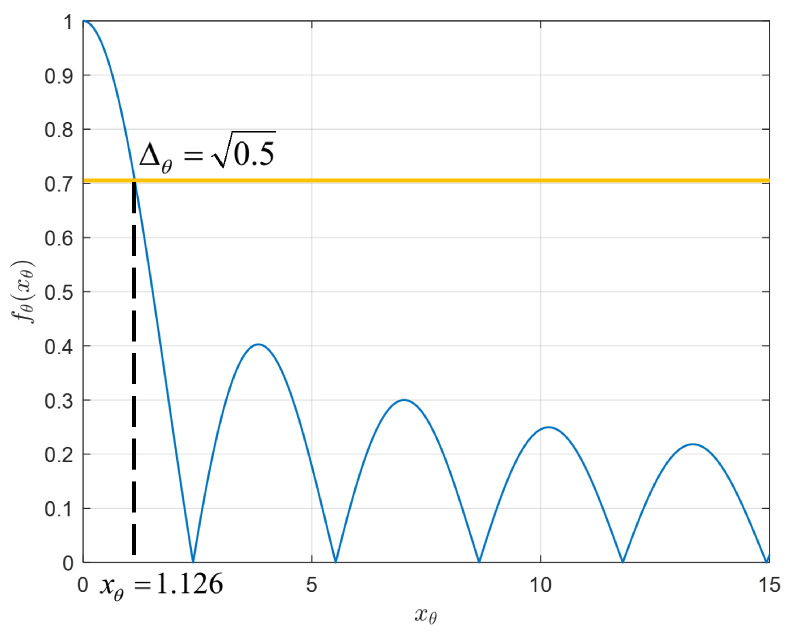
The numerical results of $f_{\theta }\left(x_{\theta }\right)$ against $x_{\theta }$.

For θ domain, the codebook is determined according to Function (14). Specifically, Figure 5 plots the numerical results of $f_{\theta }\left(x_{\theta }\right)$ against $x_{\theta }$:

It can be seen that the Bessel function achieves maximum when $x_{\theta }=0$, which means $\theta_{2}=\theta_{1}$, i.e., two ellipsoids overlap and degenerate to a point. In addition, $f_{\theta }\left(x_{\theta }\right)$ is decreasing with swings with the increase in $x_{\theta }$. Therefore, it is essential to acquire accurate angles to perform effective beamforming; otherwise, the received signal may dramatically reduce or even approach zero.

Here, we introduce $\Delta_{\theta }$ to represent the beam coherence in the θ domain. By adjusting $\Delta_{\theta }$, codebooks with different steering vector coherence can be generated. Specifically, a larger $\Delta_{\theta }$ results in a larger beam ellipsoid volume and a smaller codebook size. Assuming a 3 dB power threshold for one beam in the focusing direction of another beam, based on the relationship between $f_{\theta }\left(x_{\theta }\right)$ and signal power in this paper. So we set $f_{\theta }\left(x_{\theta }\right)=\Delta_{\theta }=\sqrt{0.5}$ in Function (14) and calculate $x_{\theta }$.

**Figure 6:**
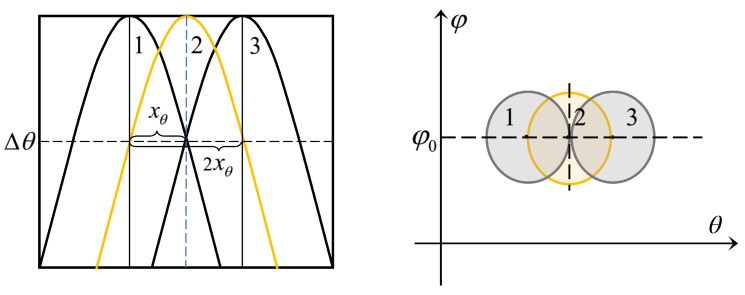
Beam tangent illustration of TAA in θ−φ domain.

It is worth noting that if the beam ellipsoid is required to be tangent, the next beam tangent to it should be $2x_{\theta }$, rather than $x_{\theta }$. In Figure 6, a beam tangent illustration of TAA in θ−φ domain is given to explain the relationship of the tangent ellipsoid and $x_{\theta }$.

We can find that the 3 dB boundary of beam 1 corresponds to beam 2 in yellow, and the tangent ellipsoid of beam 1 is beam 3.

Therefore, our proposed $\theta_{D}$-selectable set is given by:

$$2x_{\theta }=\frac{2\pi f}{c}r_{0}\left(\sin\theta_{2}-\sin\theta_{1}\right)$$

i.e.,

(21) $$\sin\theta_{n}=\sin\theta_{1}+\frac{fx_{\theta }}{\pi cr_{0}}\left(n-1\right)$$

where $\theta_{1}=\theta_{min}$, and n=1,2,⋯,N. The size of $\theta_{D}$-selectable set is:

(22) $$N=\left\lceil \frac{\pi cr_{0}}{fx_{\theta }}\left(\sin\theta_{max}-\sin\theta_{min}\right)+1\right\rceil$$

We can derive $\theta_{D}$-selectable set with the minimum power of 3 dB while $x_{\theta }=1.126$ and $f\left(1.126\right)=\sqrt{0.5}$ as shown in Figure 5.

**Figure 7:**
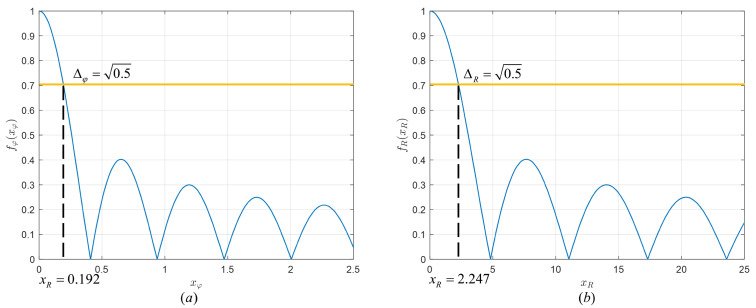
The numerical results with the same θ and *f* of (**a**) $f_{\varphi }\left(x_{\varphi }\right)$ against $x_{\varphi }$. (**b**) $f_{R}\left(x_{R}\right)$ against $x_{R}$.

In the same way, Figure 7 plots the numerical results with the same θ and *f* of $f_{\varphi }\left(x_{\varphi }\right)$ against $x_{\varphi }$ and $f_{R}\left(x_{R}\right)$ against $x_{R}$.

We can see that Functions (17) and (20) have similar properties to the Bessel function (Function (15)) in that get the maximum value at zero and decrease with swings with the increase in the independent variable.

For φ domain, the width of ellipsoids with the same θ are equal. In the same way, let $f_{\varphi }\left(x_{\varphi }^{n}\right)=\Delta_{\varphi }$, then our proposed $\varphi_{D}$-selectable set of different $\theta_{n}$ is given by:

(23) $$\varphi_{s}^{n}=2\left(s-1\right)x_{\varphi }^{n}$$

where $s=1,2,\cdots ,S_{n}$ and $S_{n}$ denotes the size of $\varphi_{D}$-selectable set in $\theta_{n}$, i.e., $S_{n}=\left\lceil \frac{\pi }{x_{\varphi }^{n}}\right\rceil$.

For *R* domain, let $f_{R}\left(x_{R}^{n}\right)=\Delta_{R}$, then $\frac{\pi f}{c}r_{0}^{2}\left(\frac{1}{R_{1}}-\frac{1}{R_{2}}\right)\sin^{2}\theta_{n}\geq x_{R}^{n}$, we have:

(24) $$\frac{1}{R_{1}}-\frac{1}{R_{2}}\geq \frac{c}{\pi fr_{0}^{2}\sin^{2}\theta_{n}}x_{R}^{n}$$

where $p=1,2,\cdots ,P_{n}$.

To make the *R*-coherence $f_{R}\left(x_{R}^{n}\right)$ lower than a given threshold, it is clear from inequality (24) that the difference between the inverses of two distances should be larger than a constant. According to [16], our proposed $R_{D}$-selectable set is given by

(25) $$R_{p}^{n}=\frac{1}{p}\frac{\pi fr_{0}^{2}\sin^{2}\theta_{n}}{cx_{R}^{n}}$$

The size of $R_{D}$-selectable set in $\theta_{n}$ is:

(26) $$P_{n}=\left\lfloor \frac{1}{R_{min}}\frac{\pi fr_{0}^{2}\sin^{2}\theta_{n}}{cx_{R}^{n}}\right\rfloor -\left\lceil \frac{1}{R_{max}}\frac{\pi fr_{0}^{2}\sin^{2}\theta_{n}}{cx_{R}^{n}}\right\rceil$$

The proposed TAA codebook design scheme is summarized in Algorithm 1.
**Algorithm 1** TAA codebook design procedure**Input:** The target serving zone, $R\in \left[R_{min},R_{max}\right],\theta \in \left[\theta_{min},\theta_{max}\right],\varphi \in \left[0,2\pi \right]$; Threshold $\Delta_{\theta },\Delta_{\varphi },\Delta_{R}$; Antenna number *M*; Operation frequency *f*; UCA radius $r_{0}$; **Output:** TAA codebook W;
1:Calculate $x_{\theta }$ accroding to (14) and $f_{\theta }\left(x_{\theta }\right)=\Delta_{\theta }$;2:Calculate the number of $\theta_{D}$-selectable set *N* according to (22);3:**for** n=1,2,⋯,N **do**4: Select $\theta_{n}$ according to (21), i.e., $\theta_{n}=\arcsin\sin\theta_{1}+\frac{fx_{\theta }}{\pi cr_{0}}\left(n-1\right)$;5: Calculate $x_{\varphi }^{n}$ according to (17) and $f_{\varphi }\left(x_{\varphi }^{n}\right)=\Delta_{\varphi }$;6: Calculate $x_{R}^{n}$ according to (20) and $f_{R}\left(x_{R}^{n}\right)=\Delta_{R}$;7: Calculate the number of $\varphi_{D}$-selectable set of $\theta_{n}$: $S_{n}=\left\lceil \frac{\pi }{x_{\varphi }^{n}}\right\rceil$, and $R_{D}$-selectable set of $\theta_{n}$ according to (26);8: **for** $s=1,2,\cdots ,S_{n}$ **do**9:  Select $\varphi_{s}^{n}$ according to (23);10:  **for** $p=1,2,\cdots ,P_{n}$ **do**11:   Select $R_{p}^{n}$ according to (25);12:  **end for**13: **end for**14: $W_{n}={a(f;R_{p}^{n},\theta_{n},\varphi_{s}^{n})}_{s=1,2,\cdots ,S_{n};p=1,2,\cdots ,P_{n}}$;15:**end for**16:$W=[W_{1},\cdots ,W_{N}]$;17:**return** W

## 5. Multi-Beam Training

Hybrid beamformers provide multi-beam capability with a reasonable compromise between performance and complexity, which can significantly accelerate beam training in the near-field scenario. In this section, we consider a multi-beam training scheme by applying a hybrid beamformer with $N_{RF}$ RF chains.

**Figure 8:**
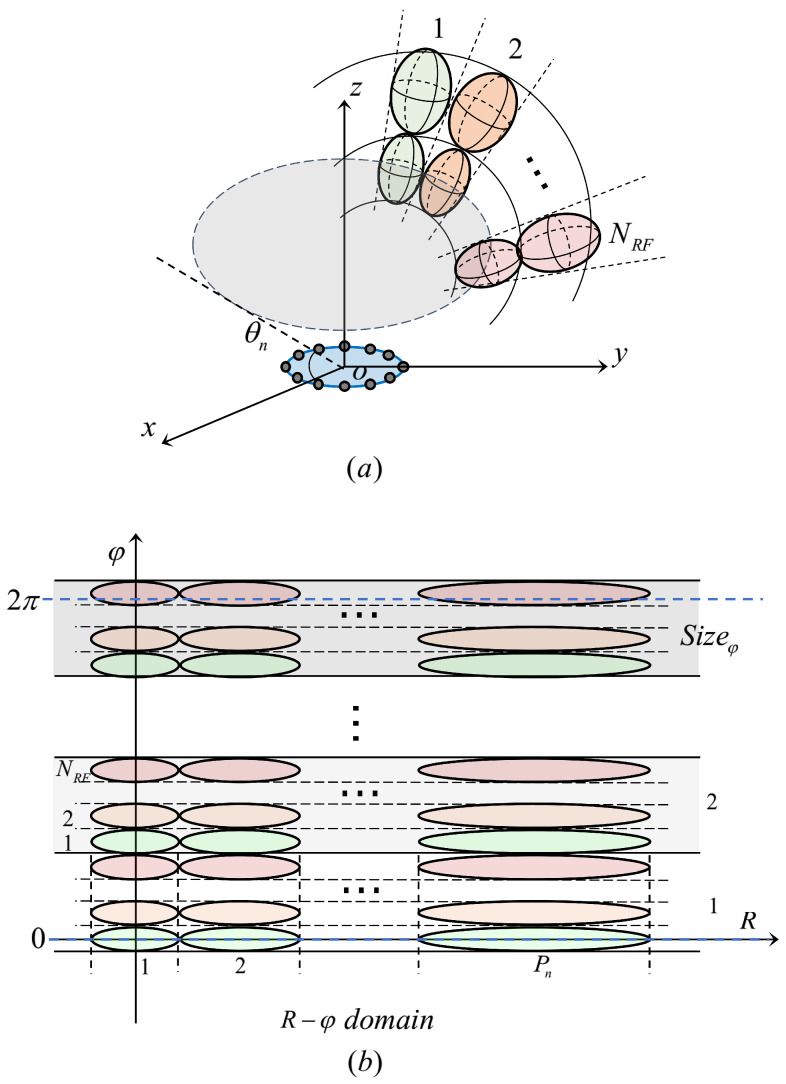
Illustration of multi-beam training scheme (**a**) in 3D view. (**b**) in R−φ domain.

To ensure that the beams can scan the whole communication serving zone, we need to generate multi-beams with certain rules to perform scanning. Owing to the $\varphi_{D}$-selectable set being an arithmetic sequence and only related to $\theta_{n}$, we divide the φ domain for multi-beam scanning. As shown in Figure 8 as below:

For the beams with the same θ, the beams on different φ have the same $R_{D}$-selectable set. From $\theta =\theta_{1}$ to $\theta =\theta_{N}$, scan the region in order. The $N_{RF}$ beams on $\theta_{n}$ generated simultaneously have the same $R_{D}$. The steps of simple multi-beam training are summarized as below:

1. For $\theta_{n}$, $N_{RF}$ beams are tangentially arranged, scanning layer by layer from $R=R_{1}^{n}$ to $R=R_{P_{n}}^{n}$.
2. Repeat the step 1 $Size_{\varphi }$ times to scan all the areas of $\theta =\theta_{n}$, where $Size_{\varphi }=\left\lceil \frac{S_{n}}{N_{RF}}\right\rceil$
3. Repeat step 1 and 2 *N* times to scan the whole target serving zone.

According to Section 4.2, the codebook size can be written as

(27) $$Size=\sum_{n=1}^{N}S_{n}P_{n}$$

We define the beam switching time $T_{s}$, so the time cost of single-beam training is

(28) $$Cost_{single-beam}=\sum_{n=1}^{N}S_{n}P_{n}T_{s}$$

The time cost of our proposed simple multi-beam training is

(29) $$Cost_{multi-beam}=\sum_{n=1}^{N}\left\lceil \frac{S_{n}}{N_{RF}}\right\rceil P_{n}T_{s}$$

## 6. Simulation Results

In this section, the simulated near-field beam patterns are plotted, and the numerical results of our proposed multi-beam training scheme are presented as well as compared with benchmarks [16,20].

**Figure 9:**
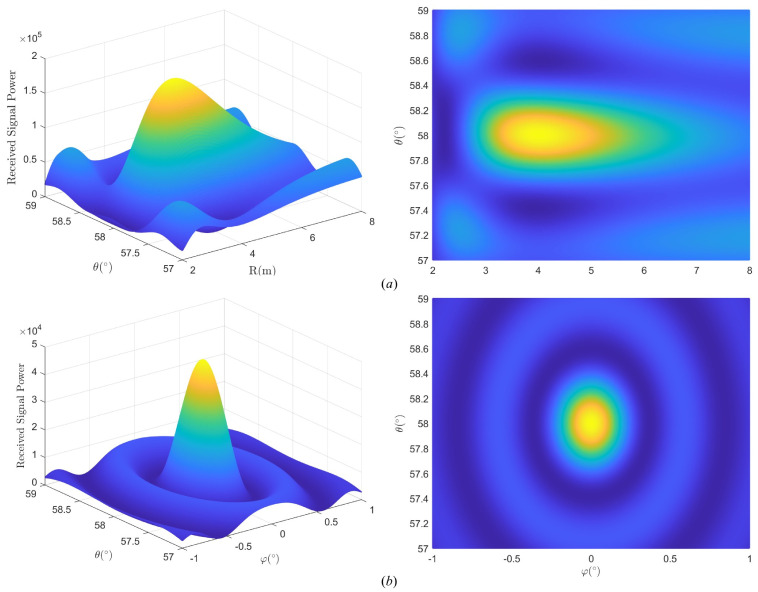
Beampattern in (**a**) R−θ domain. (**b**) φ−θ domain.

Figure 9 plots the real single-beam pattern in R−θ domain and φ−θ domain with location $\left(4\;m,58^{\circ },0^{\circ }\right)$. The system parameters are setted by $r_{0}=0.15\;m$, M=128 and f=0.14THz.

The signal power decreases and diffuses from the target user to its surroundings. We can see that the geometric shape of the near-field beam pattern of UCA is approximately an ellipsoid which is very different from the far-field region. The real beam pattern is more like a water drop, with elongating in the *R* domain.

**Figure 10:**
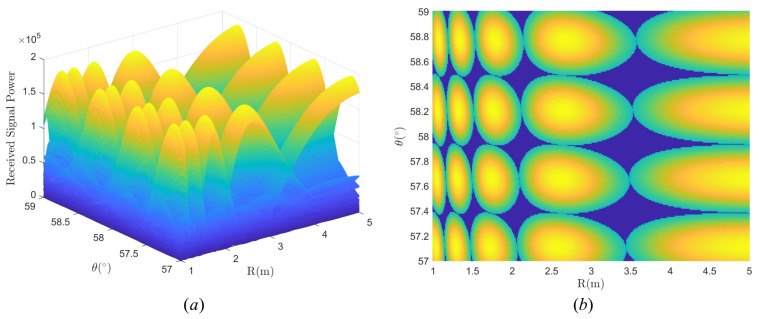
Examples of the beams in R−θ domain. (**a**) Full view by the proposed codebook. (**b**) Top view by the proposed codebook.

Considering a user’s area with [1m,40m] in distance, $\left[0^{\circ },60^{\circ }\right]$ in elevation, and $\varphi =0^{\circ }$, we obtain a codebook based on TAA. Figure 10 plots an example of the beams in R−θ domain by the proposed codebook.

One can see that the beams in different locations have different geometric shapes, and each ellipse is tangent to the surrounding ellipses. We define the coverage area by the 3 dB boundary of the beams. Since the boundary of the beam is ellipsoid, there will be coverage blind areas even if the arrangement is close, i.e., the dark blue region in Figure 10b. It is worth noting that in the blue region, the beam gain is slightly less than 3 dB but not zero. So by arranging the beams densely, it can be thought that the whole serving zone has been covered by all the beams of the codewords in our proposed codebook. If the gain in the blue region does not meet the system requirement, we can change the power threshold, for example, from 3 dB loss to 2 dB loss, and generate a codebook with higher gain by TAA.

**Figure 11:**
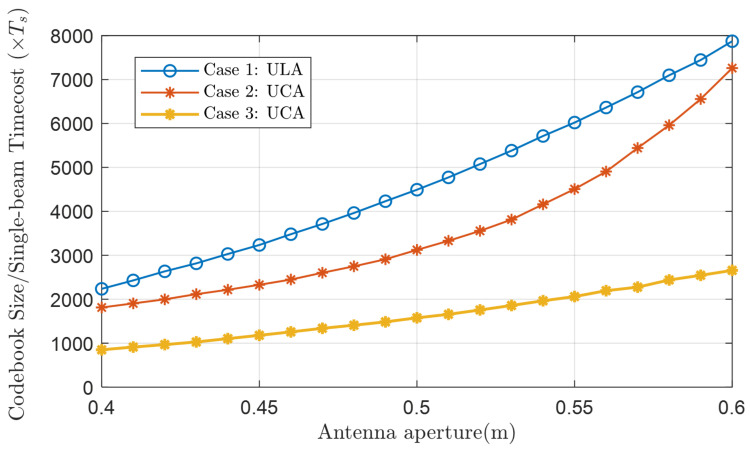
Comparison of codebook size/single-beam training time cost.

The speed of beam training often depends on the number of beams that can cover the whole target area, that is, codebook size. Figure 11 compares the codebook size, i.e., the time cost of single-beam training in R−θ domain with [1m,40m] in distance and $\left[0^{\circ },60^{\circ }\right]$ in elevation, of three cases as shown below.

- Case 1: Near-field polar-domain scheme of ULA [16].
- Case 2: Geometric beam-shape scheme of UCA [20].
- Case 3: Tangent arrangement approach of UCA (our proposed scheme).

This comparison is fair because the performance of the three cases is comparable in the 3 dB boundary, and our goal is to cover the whole communication serving zone as much as possible with the smallest codebook size.

We can see that applying UCA (Case 2 and Case 3) can significantly reduce the codebook size compared with ULA (Case 1). Ref. [20] points out that commonly the serving range is at the front of the array with $\theta \in [-\theta_{max},\theta_{max}]$. The near-field beam pattern tends to be a far-field one when the elevation angle $\theta_{D}$ tends to be π/2 for ULA, whereas $\theta_{D}$ tends to be 0 for UCA. Obviously, for the same serving zone, the codebook size of UCA will be smaller than that of ULA, i.e., the single-beam training in UCA is faster than that in ULA.

Moreover, the UCA codebook design scheme of [20] is not as accurate as our proposed scheme as there will be more overlap between ellipsoids. So the codebook size is smaller in our proposed mscheme.

**Figure 12:**
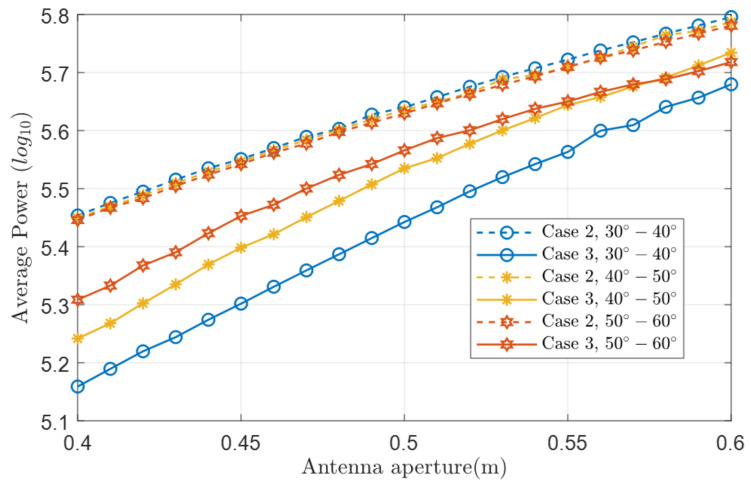
The average received power comparison of Case 2 and Case 3.

Although we achieve the coverage of the whole communication serving zone at the minimum codebook size, there is a certain loss of channel capacity, i.e., lower average received power. Figure 12 plots the average received power comparison without channel transmission in different θ regions of Case 2 and Case 3.

One can see that the received power of Case 3 is greatly affected by different θ regions, and the overall average power is lower than Case 2. The larger the antenna aperture is, the greater the average received power in case 3 is. When the smaller antenna aperture and θ region are closer to $0^{\circ }$, a higher receiving power threshold, i.e., $\Delta_{R},\Delta_{\theta },\Delta_{\varphi }$, can be chosen to improve the channel capacity.

**Figure 13:**
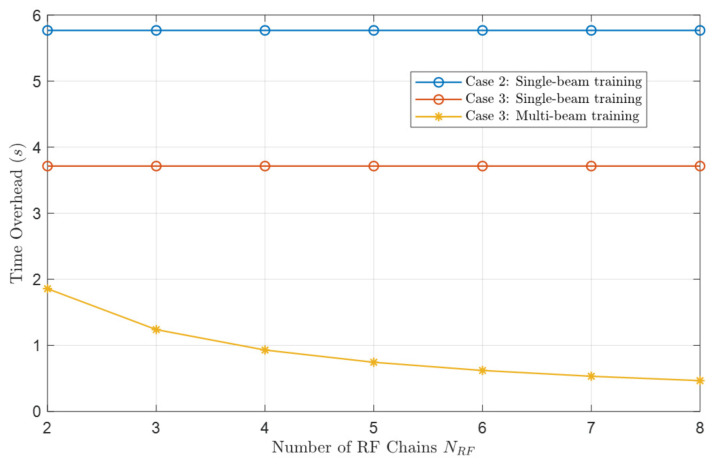
Time overhead comparison of single-beam training and multi-beam training.

To prove the superiority of our proposed multi-beam training scheme, we compare the time cost under 3 situations: single-beam training based on Case 2, single-beam training based on Case 3, and our proposed multi-beam training scheme on Case 3. Considering a user’s area with [1m,40m] in distance, $\left[30^{\circ },60^{\circ }\right]$ in elevation, and $\varphi =0^{\circ }$, the system parameters are setted by the frequency f=0.14 THz, the radius of UCA $r_{0}=0.15$ m, antenna number M=128, and the beam switching time $T_{s}=1$ µs. The simulation result is shown in Figure 13.

The codebook size in Case 2 is 576,771, and 371,409 in Case 3. Applying the TAA saves nearly one-third of the time overhead of beam training compared to Case 2. In addition, our proposed multi-beam training scheme can further accelerate beam training while bringing hardware costs to RF chains.

## 7. Conclusions

In this paper, we considered a THz UCA with hybrid beamforming architecture in a near-field communication scenario. By analyzing the geometric beam shape of the near-field UCA, we proposed a near-field codebook by analyzing different beams’ coherence θ,φ, and *R* domain. Specifically, we found that the coherence function of θ domain conforms to the zero-order Bessel function of the first kind, and the coherence function of φ domain and *R* domain have similar properties to the Bessel function, which helps us to design the codebook according to a closed-form solution. To boost the training speed, we proposed a multi-beam training scheme by using the hybrid beamforming architecture. Numerical results showed the effectiveness and superiority of our proposed codebook compared to the benchmarks, as it incurs less training time with comparable coverage performance.

## Figures and Tables

**Table 1:** Relationship Between the Beam Pattern and Target Point [20].

Moving Target Point	Length ΔR	Width Δθ	Thickness Δφ
$\left(R_{D}\;↑,\theta_{D},\varphi_{D}\right)$	↑	-	-
$\left(R_{D},\theta_{D}\;↑,\varphi_{D}\right)$	↓	↑	↓
$\left(R_{D},\theta_{D},\varphi_{D}\;↑\right)$	-	-	-

## Data Availability

Not applicable.

## Abbreviations

The following abbreviations are used in this manuscript:

THz	Terahertz
BS	Base Station
UCA	Uniform Circular Array
TAA	Tangent Arrangement Approach
6G	Six-Generation
AR	Augmented Reality
VR	Visual Reality
ULA	Uniform Linear Array
LoS	Line-of-Sight
UM-MIMO	Ultra-Massive Multiple-Input-Multiple-Output
CSI	Channel State Information
IO	Input/Output
RF	Radio Frequency
3D	Three Dimensional
